# Metabolic Engineering of the Native Monoterpene Pathway in Spearmint for Production of Heterologous Monoterpenes Reveals Complex Metabolism and Pathway Interactions

**DOI:** 10.3390/ijms21176164

**Published:** 2020-08-26

**Authors:** Chunhong Li, Sreelatha Sarangapani, Qian Wang, Kumar Nadimuthu, Rajani Sarojam

**Affiliations:** 1 Temasek Life Sciences Laboratory, 1 Research Link, National University of Singapore, Singapore 117604, Singapore; chunhong@tll.org.sg (C.L.); sreelatha@tll.org.sg (S.S.); kumarnadi@tll.org.sg (K.N.); 2 College of Animal Science, Zhejiang University, Hangzhou 310029, China; Emirate14@zju.edu.cn

**Keywords:** limonene synthase, terpenes, secondary metabolites, metabolic engineering, pathway flux analyses, derivatives

## Abstract

Spearmint produces and stores large amounts of monoterpenes, mainly limonene and carvone, in glandular trichomes and is the major natural source of these compounds. Towards producing heterologous monoterpenes in spearmint, we first reduced the flux into the native limonene pathway by knocking down the expression of limonene synthase (*MsLS*) by RNAi method. The *MsLS* RNAi lines exhibited a huge reduction in the synthesis of limonene and carvone. Detailed GC-MS and LC-MS analysis revealed that *MsLS* RNAi plants also showed an increase in sesquiterpene, phytosterols, fatty acids, flavonoids, and phenolic metabolites, suggesting an interaction between the MEP, MVA shikimate and fatty acid pathways in spearmint. Three different heterologous monoterpene synthases namely, linalool synthase and myrcene synthase from *Picea abies* and geraniol synthase from *Cananga odorata* were cloned and introduced independently into the *MsLS* RNAi mutant background. The expression of these heterologous terpene synthases resulted mainly in production of monoterpene derivatives. Of all the introduced monoterpenes geraniol showed the maximum number of derivatives. Our results provide new insights into MEP pathway interactions and regulation and reveals the existence of mechanisms for complex metabolism of monoterpenes in spearmint.

## 1. Introduction

Terpenes/or terpenoids constitutes the largest class of plant secondary metabolites with diverse biological functions in plants. They are important for plants defense responses, plant to plant communication and plant pollinator attraction [1]. More than 55,000 terpenoid structures have been identified [2]. Terpenes also have enormous commercial value because of their applications in food, cosmetic, agricultural, and pharmaceutical industries [3,4]. The precursor of all different types of terpenes are the five carbon isoprene units, isopentenyl diphosphate (IPP) and its isomer dimethylallyl diphosphate (DMAPP). In plants, they are synthesized from two different pathways localized in different subcellular compartment. The mevalonate (MVA) pathway in the cytoplasm generates precursors towards the formation of sesquiterpenes and triterpenes and the 2-*C*-methyl-d-erythritol 4-phosphate (MEP) pathway in plastids produces precursors that are responsible for the production of monoterpenes, diterpenes and tetraterpenes. Evidence of cross talk between the MVA and MEP pathway has been observed in *Arabidopsis*, tobacco and *Antirrhinum* [5,6,7]. Among the terpenoids, monoterpenoids (C_10_) and sesquiterpenoids (C_15_) are volatile terpenoids and together they form the largest class of plant volatile organic compounds (VOCs). They are commonly found as components of natural flavors and fragrances. Monoterpenes produced by the plant can either be stored in specialized organs like glandular trichomes or emitted into the plant head space. Isopentenyl diphosphate (IPP) and dimethylallyl diphosphate (DMAPP) generated by MEP pathway are condensed together by geranyldiphosphate synthase (GDPS) to produce geranyldiphosphate (GDP) which is the precursor of all monoterpenes. Monoterpene synthases (TPS) are the enzymes responsible for synthesis of monoterpene parent scaffold from the precursor GDP. Given the ecological and commercial value of monoterpenes, attempts towards pathway engineering have been made in native or heterologous plants for altering floral scent profiles, fruit flavors, diversifying plant’s biotic and abiotic interactions and also for enhanced production of valued VOCs [8,9].

Monoterpenes such as linalool, geraniol, and myrcene are major constituents of floral scent and fruit flavors. They also find wide usage in flavor and fragrance, agriculture, and pharma industries. Geraniol and linalool are acyclic monoterpenoid alcohol synthesized from GDP by geraniol synthase (GES) and linalool synthase (LS), respectively. Myrcene is a linear acyclic monoterpene synthesized by myrcene synthase. Heterologous expression of GES in tobacco/tobacco hairy roots, *Arabidopsis*, maize, and vitis have been reported [10,11,12,13]. High production of linalool has been attempted by overexpression of linalool synthase (LS) in *Lavandula, petunia*, *Arabidopsis, Chrysanthemum*, clarkia and tomato [5,14,15,16,17]. 

These studies showed quantitative and qualitative differences in the production of geraniol/ linalool and its derivatives in different plant backgrounds. This highlighted the fact that the fate of an expressed monoterpene is species specific and depends on the endogenous modifying enzymes present in the plant cell. Lately *Nicotiana benthamiana*, is emerging as a popular heterologous plant expression platform for production of plant metabolites, including monoterpenes. This is largely due to its amenability to agrobacterium-mediated transformation techniques allowing rapid expression of several metabolic pathway genes [18]. Initial attempt to produce heterologous monoterpenes in tobacco could only generate small amounts [8]. For instance, transgenic tobacco overexpressing terpinene synthase, (+)-limonene synthase and β-pinene synthase from lemon (*Citrus limon*) showed trace amounts of emission from leaves and flowers [19]. Later, studies reported success in enhancing the production of monoterpenes by regulating the compartment localization of terpene synthase [1,11,20]. Further co-expression of a GDP synthases along with a monoterpene synthase, geraniol synthase, in different subcellular compartment led to higher accumulation of free geraniol and its glycoside derivates in plastid, cytosol and mitochondria [1]. Later it was reported that overexpression of pepper mint geranyl diphosphate synthase small subunit (MpGPS.SSU) could enhance the production of various monoterpenes such as (–)-limonene, (–)-linalool, (–)-α-pinene/β-pinene or myrcene in transient and stable transgenic tobacco [21]. 

Tobacco by nature does not produce monoterpenes and lacks structures like glandular trichomes for storing such volatiles. Aromatic plants of genus *Mentha*, a member of the family Lamiaceae, which includes well known species like *M. piperita* (peppermint), and *M. spicata* (spearmint) are known to produce plant volatiles in specialized structures called peltate glandular trichomes (PGT). Mentha plants are routinely used for commercial scale recovery of plant terpenes. The PGT are non -photosynthetic organs, solely committed to the production and storage of large amounts of volatile secretions [22,23,24]. Spearmint PGT consists of a basal cell, a stalk cell, and eight secretory cells [25] and it produces essential oil dominated by two monoterpenes, limonene and carvone. Biosynthetic pathway for limonene and carvone production is well characterized. First, GDP is cyclized by a terpene synthase known as limonene synthase (LS) to (–)-limonene, which is then converted to trans-carveol by limonene-6-hydroxylase, and then dehydrogenated to (–)-carvone by trans carveol dehydrogenase [26]. Limonene synthase gene has been cloned and characterized from spearmint. In vitro study has shown that MsLS mainly produces limonene from GDP along with small amounts of alpha- and beta-pinene and myrcene [27,28]. Attempts have been made to increase the native monoterpene content in transgenic *Mentha* either by manipulating genes that code for pathway enzymes or by perturbing transcription factors controlling the pathway [29,30,31,32,33].

In this study, we evaluated the potential of spearmint for production of heterologous monoterpenes. Towards this, we first reduced the flux into the native limonene pathway by knocking down the expression of limonene synthase (*MsLS*) by RNAi method. The *MsLS* RNAi lines exhibited huge reduction in the synthesis of limonene and carvone. Detailed GC-MS and LC-MS analysis revealed that *MsLS* RNAi plants also showed an increase in metabolites derived from MVA, phenylpropanoid and fatty acids pathway suggesting an interaction between these pathways in spearmint. Towards producing heterologous monoterpenes, three previously characterized monoterpene synthases linalool synthase (PaLinS) and myrcene synthase (PaMyrS) from *Picea abies* and geraniol synthase (CoGerS) from *Cananga odorata* (ylang-ylang) were cloned and introduced independently into the *MsLS* RNAi mutant background [21,34]. The expression of the heterologous terpene synthases resulted mainly in production of respective monoterpene derivatives. Our results provide new insights into MEP pathway interactions and regulation in spearmint and reveals the existence of mechanisms for complex metabolism of monoterpenes. This will help towards developing improved strategies for metabolic engineering of the monoterpene pathway for biological as well as commercial purposes and selection of suitable plant chassis.

## 2. Results

### 2.1. Generation of RNAi Mediated Knock Down Line of Limonene Synthase Gene in Spearmint

**Figure 1:**
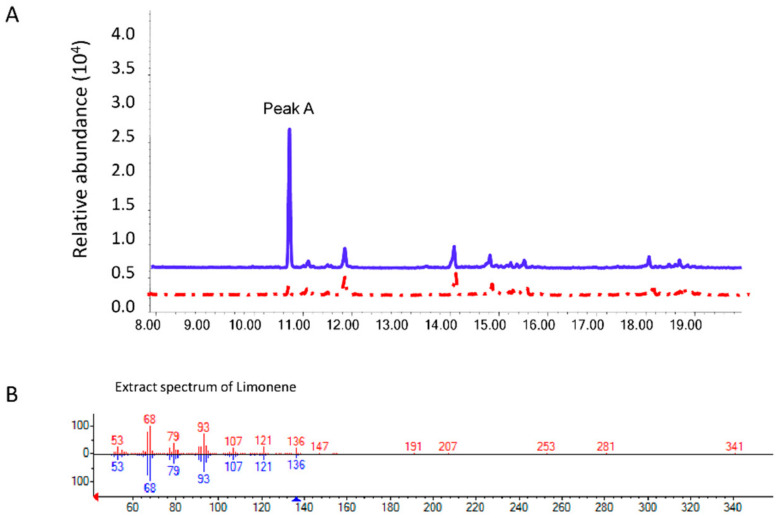
Functional characterization of recombinant MsLS in vitro (**A**) GC-MS profile of recombinant MsLS protein activity with (in blue) and without GDP (red). (**B**) Mass spectrum of peak A compared to the matched limonene peak from NIST library.

We identified and cloned the limonene synthase gene *MsLS* from the previously published RNA seq data of spearmint PGT from our lab [35]. Full length ORF of *MsLS* was cloned from mint PGT cDNA and found 98 percent similar to the previously reported LS gene (L13459) (Figure S1) [27]. The gene was functionally characterized in vitro to test its ability to produce limonene from GDP. *MsLS* was cloned into pDEST-6His using gateway cloning system and expressed in *E. coli* BL21(DE3). Recombinant MsLS was purified using HisPurTM Ni-NTA spin purification kit (ThermoFisher Scientific, Singapore) The purified protein was then used to assay for production of limonene from GDP in vitro. The products were analyzed by GC-MS based on a comparison of retention times and mass spectra of limonene standard. Results showed that incubation of MsLS with GDP produced limonene (Figure 1A). 

**Figure 2:**
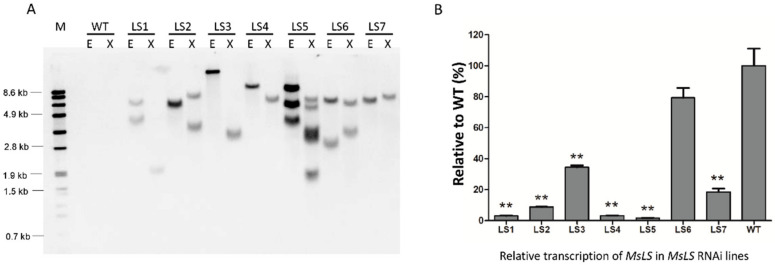
Characterization of MsLS RNAi lines (**A**) Southern blot analysis of MsLS RNAi lines. DNA from transgenic plants and wild type (WT) were digested with EcoRI (E) and Xbal (X). M indicates DNA Molecular Weight Marker Vii. (**B**) MsLS transcripts level analysis by qRT-PCR. Gene expression is presented as relative to that of WT (%). Data represent as mean ± SD for three biological replicates. (**, *p* ≤ 0.01).

No terpenes were detected when MsLS was incubated with farnesylpyrophosphate (FPP) and geranylgeranyl diphosphate (GGPP). With the aim to reduce flux into the native limonene biosynthesis pathway, *MsLS* RNAi lines were generated. Of the seven independent transgenic lines confirmed by southern blot, three had single insertion of T-DNA (Figure 2A). Six out of the seven lines showed a significant reduction in *MsLS* expression when compared to WT ranging from 65 to 98% as determined by qRT-PCR (Figure 2B). No phenotypic difference was observed in the *MsLS* RNAi lines and they appeared similar to wild type plants. The comparison of MsLS RNAi lines and WT was based on observable characteristic of both the plants, such as plant height, leaf size and shape, growth rate etc.

### 2.2. Silencing of MsLS Reduces Monoterpene Production and Affects Flux into Other Metabolic Pathways

Three lines (2,4 and 5) with significant reduction in the expression of *MsLS* were chosen for further study. Single quadrupole GC-MS analysis was performed on young leaves (2–3 cm) to analyze the effect on production of monoterpenes upon *MsLS* suppression. 

**Figure 3:**
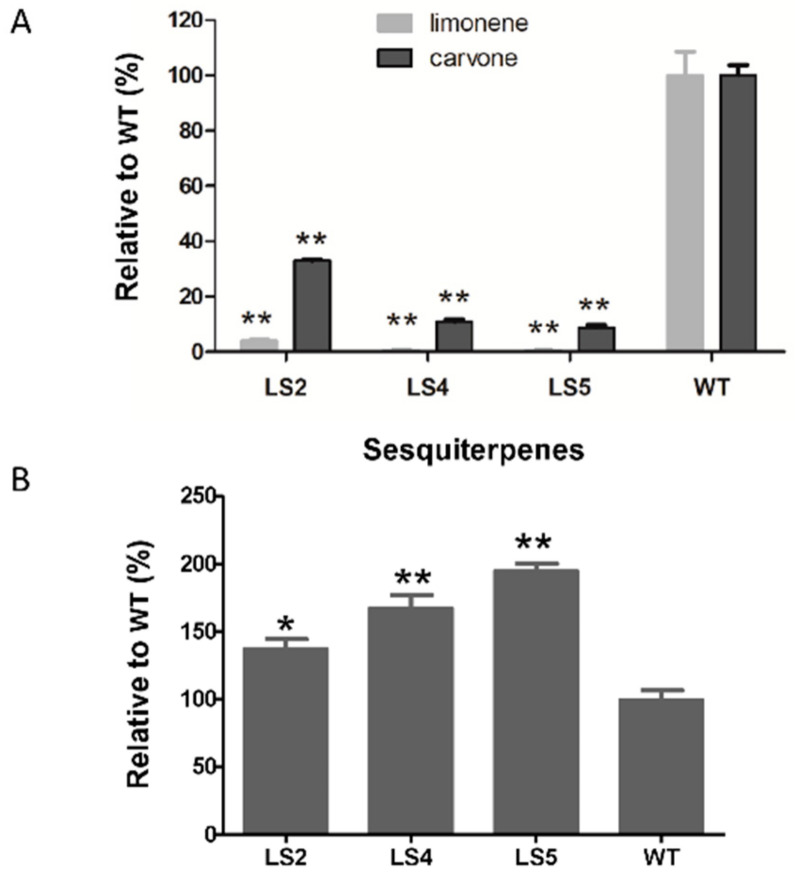
Volatile component analysis of three MsLS RNAi plant (LS2, LS4 and LS5) and wild type (WT) by GC-MS (**A**) Quantitative analysis of two major monoterpenes. (**B**) Quantitative analysis of sesquiterpenes. Amount of terpenes production are presented as relative to that of WT. Data represent as mean ± SD for three biological replicates. (*, *p* ≤ 0.05; **, *p* ≤ 0.01).

Peaks were identified based on comparison of the mass spectra with authentic standards. All the three lines exhibited substantial decrease in both limonene and carvone production, ranging from 96% to 99% for limonene and 67% to 91% for carvone, when compared to WT respectively (Figure 3A). Although the MEP and MVA pathway are well separated there are studies that indicate a crosstalk between the two pathways. The MVA pathway is mainly responsible for the formation of sesquiterpenes and phytosterols. To identify changes in metabolites on a larger scale an untargeted GC- and LC-QTOF-MS analysis was performed on the three *MsLS* RNAi lines. 

**Figure 4:**
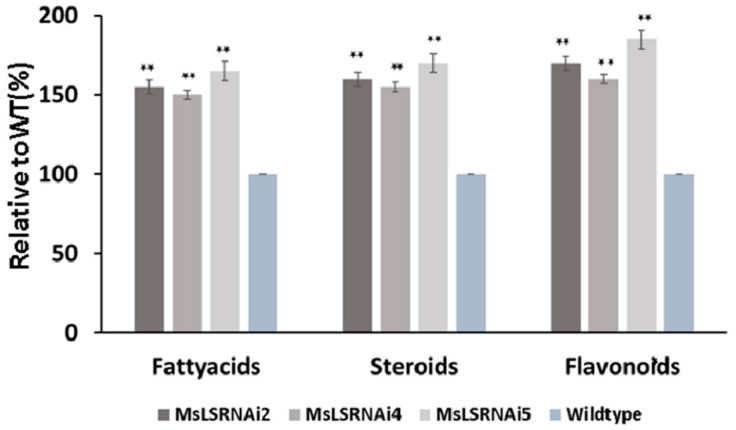
Percent composition pf fatty acids, sterroids, and flavonoids in wildtype and MsLS RNAi lines using GC-MS and LC-MS analysis. Data represent mean ± SD for three biological replicates. (**, *p* ≤ 0.01).

The major sesquiterpene observed in the spearmint variety are caryophyllene, *cis*-β-farnesene, *cis*-muurola-4(15), 5-diene and germacrene D. Single quadrupole GC-MS analysis revealed that all the three *MsLS* RNAi lines showed increase in total amount of sesquiterpenes ranging from 38% to 96% when compared to WT (Figure 3B). Additionally, GC-QTOF-MS showed an increase in phytosterols namely stigmasterol and sitosterol ranging from 55% to 65% when compared to WT (Figure 4). An increase in sesquiterpenes and phytosterols indicate an increase in flux towards the MVA pathway upon suppression of the native monoterpene pathway. Further GC and LC–QTOF-MS analysis, showed an increase in the total amounts of fatty acids ranging from 40 % to 55 %, and in flavonoids and phenolic metabolites ranging from 65% to 85 % in all the three *MsLS* RNAi lines when compared to WT respectively (Figure 4 and Figure S2). Table 1 and Table 2 list the various metabolites, which were found significantly increased upon *MsLS* suppression.

The MEP pathway is localized in the plastids, which is also the site for shikimate and fatty acid pathways. All the three pathways share phosphoenolpyruvate and pyruvate as common biosynthetic precursors derived from primary metabolism. Our results indicate that alteration of MEP pathway in spearmint also affects the production of metabolites from the other two pathways present in the plastids.

### 2.3. Expression of Heterologous TPSs in MsLS RNAi Background

Among the seven *MsLS* RNAi lines, line 4 was chosen for introduction of the heterologous terpene synthases, namely *MsLS* RNAi4. *MsLS* RNAi4 was a single T-DNA insertion line and showed significant reduction in *MsLS* expression and endogenous monoterpene accumulation. The three heterozygous monoterpene synthases (TPSs), linalool synthase (PaLinS) and myrcene synthase (PaMyrS) from *Picea abies* and geraniol synthase (CoGerS) from *Cananga odorata* (ylang-ylang) were introduced into the *MsLS* RNAi4 individually. At least 6 transgenic lines were generated for each TPS. They were characterized by southern blotting and analyzed for individual TPS expression by qRT-PCR (Figures S3 and S4). Three independent single insertion lines with high TPS expression were advanced for further characterization for each TPS gene. Compared to MsLS RNAi4 with plant growth rate, plant height, leaf size and leaf shape, no observable differences were observed in analyzed transgenic lines expressing PaMyrS, CoGerS and PaLinS.

### 2.4. Metabolite Profiling of Transgenic Lines Expressing Heterologous TPSs in MsLS RNAi4 Reveals Complex Metabolism of Introduced Monoterpenes

Three independent lines expressing linalool synthase (PaLinS), myrcene synthase (PaMyrS) and geraniol synthase (CoGerS) respectively were propagated and maintained for the purpose of metabolite profiling. As monoterpenes are known to be further metabolized to nonvolatile derivatives, methanol extract of leaves of transgenic plants were also analyzed by LC–QTOF-MS in negative mode and positive mode in an untargeted approach to identify derivatives of introduced monoterpenes. 

**Figure 5:**
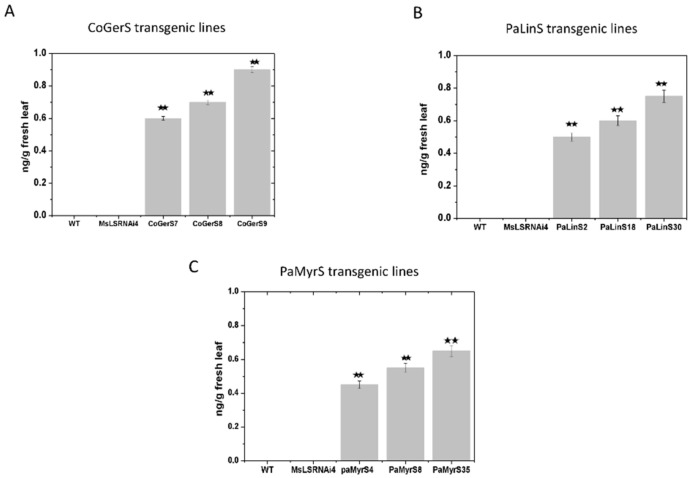
Amounts of the introduced monoterpene derivatives quantified using LC MS analysis for (**A**) CoGers transgenic lines (**B**) PaLinS transgenic lines and (**C**) PaMyrs transgenic lines respectively. Data represent mean ± SD for three biological replicates. (**, *p* ≤ 0.01).

GC-QTOF-MS analysis of three lines expressing geraniol synthase (CoGerS) revealed the presence of geraniol related compounds such geranyl isovalerate, nerolidyl propionate, geranyl hexanoate, geranyl oleate, geranyl palmitate and geranyl acetate but at low levels (up to 5% in the presence of a high sample matrix background). These compounds were not found in WT or *MsLS* RNAi lines. LC-QTOF-MS analysis revealed the presence of geraniol glycosides which were putatively identified as malonyl–hexosyl geraniol dimer, malonyl hexosyl geranic acid dimer and hexosyl hydroxy geranic acid. Loganin, an iridoid was also observed (Table 3 and Figure S5). Geraniol is a known precursor metabolite of the monoterpenoid iridoid pathway. The above data indicates towards the bioconversion of geraniol by spearmint endogenous enzymes to many different compounds. Further LC-MS/MS of geraniol standard at series of different concentrations was performed to establish a calibration curve. The total amount of geraniol derivatives was quantified based on the relative response factor. With this method the amount of total geraniol derivatives (Figure 5A) observed ranged from 0.6 ng/g to 0.85 ng/g. 

GC-QTOF-MS analysis of three lines expressing linalool synthase (PaLinS) detected no significant amount of free linalool or volatile linalool derivatives in them. LC-QTOF-MS analysis showed the presence of glycosylated non-volatile derivatives of linalool, which were putatively identified as linalool-3-glucoside, and linalyl β-d-glucopyranoside. A linalool ester was also observed which was putatively identified as linalool butyrate (Table 3 and Figure S6). Further LC-MS/MS of linalool standard at different concentrations was performed to establish a calibration curve and linalool derivatives were quantified based on the relative response. With this method the amount of total linalool derivatives (Figure 5B) observed in the three lines ranged from 0.5 ng/g to 0.8 ng/g.

**Figure 6:**
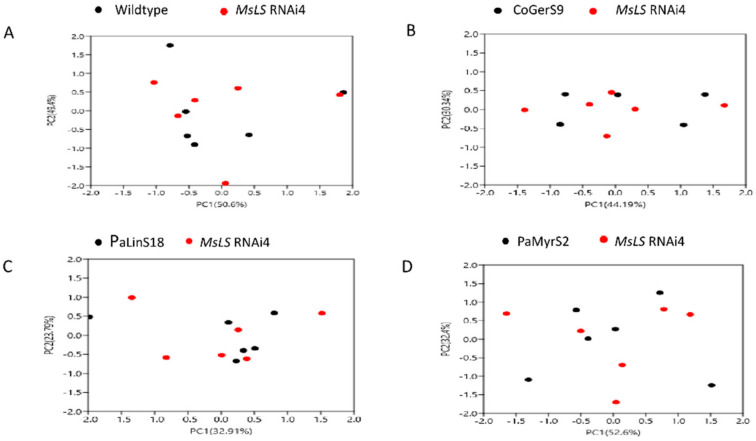
Principal component analysis for LC-MS data obtained. Score plot of (**A**) MsLS RNAi4 with wild type (**B**) MsLS RNAi4 with CoGerS9 transgenic line (**C**) MsLS RNAi4 with PaLinS18 transgenic line and (**D**) MsLS RNAi4 with PaMyrS2 transgenic line. Explained variance for PC1 and PC2 are indicated as spercentages in X and Y axis respectively.

Three lines expressing myrcene synthase were analyzed by GC-QTOF-MS but no significant amount of free myrcene or volatile myrcene derivatives were found. LC-QTOF-MS analysis showed the presence of non-volatile derivatives of myrcene which were putatively identified as myrcenyl acetate and dimethyl-3-oxopentane-1-5-diyl dibenzoate (Table 3 and Figure S7). LC-MS/MS of myrcene standard at different concentrations was performed and myrcene derivative levels were quantified based on the relative response factor of the standard and derivatives. With this method the amount of total myrcene derivatives (Figure 5C) observed in the three lines ranged from 0.4 ng/g to 0.6 ng/g. Of all the introduced monoterpenes geraniol showed the maximum number of derivatives and myrcene the least. The above results showed complex metabolism of introduced heterologous monoterpene by endogenous enzymes of spearmint. Principal component analysis was performed between WT and *MsLS* RNAi4 LC-MS data sets and between *MsLS* RNAi4 and CoGerS, PalinS and PaMyrS lines individually. The PCA (Figure 6A–D) score plot showed the distribution of the metabolites between the respective groups. The PCA analysis for WT and *MsLS* RNAi4 showed two principal components which accounted for 50.6% and 49.4% of the variation with Eigen values for PC1 (3.00) and PC2 (1.78) in the spectral data respectively. For *MsLS* RNAi4 and CoGerS line two principal components accounted for 44.1% and 30.3% of variation with Eigen values as PC1 (2.22) and PC2 (1.52). *MsLS* RNAi4 and PalinS line showed two principal components accounting for 32.9% and 23.7% variation with Eigen values as PC1 (2.43) and PC2 (1.65). For *MsLS* RNAi4 and PaMyrS line, two principal components accounted for 52.6% and 32.4% of the variation, with Eigen values are PC1 (5.06) and PC2 (3.06) respectively. Based on these results, we can now detect the distribution of metabolites whose levels are abundant between the groups. The positive values strengthen the evidence of differences between samples groups, negative values argue against. Thus, the PCA score plots showed a visible discrimination along the PC1 and PC2 explaining the variation of the data.

## 3. Discussion

Monoterpenes and their derivatives form a diverse group of plant secondary metabolites which have high ecological and economical importance. Attempts towards metabolic engineering of monoterpenes have been made to decipher this fundamental pathway in plants, to improve plant fitness with regards to stress response and to increase production of commercially valuable monoterpenes [8,36,37,38,39]. Plants like *Nicotiana benthamiana* are being engineered to serve as plant-based platforms for the sustainable biosynthesis of valuable monoterpenes which are produced in low amounts in their native plants. Microbial platforms for efficient production of monoterpenes are hampered by the issues of toxicity and volatility of these compounds. Spearmint is a fast growing, easy to cultivate plants which has glandular trichomes to store these volatile surpassing toxicity issues. Mentha plants have been previously successfully engineered for increased production of their native monoterpenes [30,32,33,40]. In this study, we suppressed the native monoterpene pathway in spearmint and explored its potential to produce heterologous monoterpenes. 

Compartmentalization of the MEP pathway in plastids and MVA pathway in cytoplasm allows plants to spatially separate biosynthesis of various types of terpenes with different functions. Initial feeding experiments with labelled precursors indicated an exchange of common intermediates between the MVA and MEP pathway [41,42,43,44]. The drawback with feeding experiments with externally supplied metabolites was that they were unable to establish the cross talk between pathways upon inhibition of specific terpene biosynthesis pathways. Later studies with mutant plants established that the extent of cross flow of intermediates between the two pathways is limited and depends upon the plant species [45,46]. In this study, limonene synthase, the major active terpene synthase in spearmint was down regulated by RNAi method to reduce flux into the native monoterpene pathway. This suppression revealed complex interaction between various metabolic pathways. An increase in the amounts of compounds derived from the MVA pathway mainly sesquiterpenes and sterols was observed. Spearmint variety used produces low levels of 4 different types of sesquiterpenes namely caryophyllene, *cis*-β-farnesene, *cis*-muurola-4(15),5-diene and germacrene. GC-MS analysis of three independent *MsLS* RNAi lines showed a 38% to 96% increase in total sesquiterpenes when compared to WT lines. The increase was not same for each metabolite. β-Sitosterol, stigmasterol and campesterol are the main phytosterols found in plants [47,48] The sterols that were found significantly increased upon *MsLS* suppression were sitosterol and stigmasterol. Pathway intermediates like IPP, DMAPP and GPP can be translocated through plastid membrane [45,46]. Suppression of limonene synthase in spearmint would possibly prevent the utilization of GPP, IPP and DMAPP into the monoterpene pathway and hence they could get channelized into the MVA pathway increasing its carbon flux. 

LC-QTOF-MS analysis also revealed an increase in the production of various flavonoids and fatty acids in the *MsLS* RNAi lines. Apart from the terpenoid MEP pathway, plastids are also the sites for shikimate and fatty acid biosynthesis [49,50,51]. All these three pathways share phosphoenolpyruvate and pyruvate as common precursors. Alteration of one pathway can affect flux into others. Shikimate pathway is responsible for generating the aromatic amino acid L-phenylalanine which serves as a precursor for phenylpropanoid pathway. This pathway generates a variety of secondary metabolites including flavonoids [52,53]. The flavonoids which were found significantly increased in amounts and the new flavonoids produced are shown in table1. Sweet basil plants like spearmint produces its essential oil in PGTs. Sweet basil essential oil is mainly composed of terpenes (monoterpenes and sesquiterpenes) and phenylpropanoids. A system biology approach to analyze the regulation of the terpene and phenylpropanoid pathway within the PGTs suggested that shikimate/phenylpropanoid and the MEP/terpenoid pathways compete for carbon and attempts to alter one pathway may influence the metabolites derived from the other [54]. Our study shows that upon suppression of MEP pathway, the flavonoid/phenolics content increases possibly due to the increase flux into shikimate/phenylpropanoid pathway. Further, fatty acid methyl esters abundance was increased upon *MsLS* inhibition. This too can be attributed to the possible increase in the flux due to perturbation in the monoterpene pathway. Additionally, in animal system a co-regulation of fatty acid production and sterol biosynthesis is observed for proper maintenance of cell membranes during cellular growth [55,56]. Studies in Arabidopsis sterol biosynthetic mutants have also revealed changes in fatty acid composition indicating a link between sterol and fatty acid metabolism [57,58,59]. The changes in fatty acid esters is solely due to increase in flux into fatty acid biosynthesis upon monoterpene pathway inhibition or increase in sterols also has an impact on FA metabolism remains to be deciphered. Taken together all these finding indicate that upon perturbation of the MEP/monoterpene pathway in spearmint PGT, the carbon flux can be redirected to other metabolic pathways active in plastids. Additionally, a cross talk between the MVA and MEP pathway is also observed. This points towards the complexity and interactions of the terpene metabolic network with other primary and secondary pathway networks. PCA is a powerful method which performs dimensionality reduction in the data set. The PCA score plot of all respective groups showed higher total mass variation along PC1 and PC2 components. The *MsLS* RNAi4 data points separated well in the positive side and these compounds positively correlated with PC1 variation indicating higher variability compared to wildtype. Overall, the variation denoted the corresponding metabolites that are responsible for the differences between the groups. Data points of *MsLS* RNAi4 expressing the heterologous TPSs separated more both in positive side and negative region indicating moderate level of metabolite variability compared to *MsLS* RNAi4. 

When heterologous terpene synthases were introduced into the *MsLS* RNAi4 background, all the three introduced monoterpenes were found converted to non-volatile derivatives. The amount of free monoterpene produced was very low. Expression of geraniol synthase (CoGerS) in heterologous hosts spearmint resulted in the formation of nonvolatile geraniol glycosides. In addition, oxidation of geraniol to geranic acid was also observed, which gets further glycosylated as well. Similar compounds were found when geraniol synthase was ectopically expressed in tobacco [1]. Geraniol is also a known precursor for iridoids which are oxidized derivatives of geraniol [60]. Loganin, an iridoid glycoside was also found in transgenic plants expressing geraniol synthase revealing the complex metabolism of geraniol by endogenous enzymes in spearmint. Analysis of transgenic lines expressing linalool synthase (PaLinS) also revealed the presence of glycosylated derivatives (linalool -3 glucoside and Linalyl β-d-glucopyranoside) and ester of linalool (Linalool butyrate). Ectopic expression of linalool synthase in Clarkia also resulted in the accumulation of linalyl-β-d-glucopyranoside which is presumed to be produced by the action of an endogenous glycosyltransferase that conjugates the linalool produced to linalyl-β-d-glucoside [9]. From the transgenic lines expressing myrcene synthase (PaMyrS) two putative myrcene derivatives, myrcenyl acetate (ester) and dimethyl-3-oxopentane-1-5-diyl dibenzoate were identified. Similar conversion of myrcene to dimethyl octane by biotransformation has been previously reported [61]. Geraniol and linalool are monoterpenes possessing an alcohol functional group whereas myrcene is a simple acyclic monoterpene. The alcohol functional group makes these compounds chemically more reactive and amenable to secondary transformations such as glycosylation, oxidation, esterification, and methylation. Among this, glycosylation is more common, and glycosylation of monoterpenes makes it non-volatile, more water soluble and decreases its toxicity [9,60]. Glycosylated monoterpenes in plants serve as a significant reservoir of volatile precursors which upon proper developmental cues or stress can be converted to active volatiles [62]. Glycosylated monoterpenes are also valued commercially. In our study we found that geraniol and linalool underwent mostly oxidation and glycosylation in spearmint. Previous studies have suggested that oxidation and glycosylation of monoterpenes in heterologous host can be a mechanism to prevent phytotoxicity of monoterpene accumulation. Spearmint produces and accumulates its volatiles in specialized organs, the PGT, which should preclude the toxicity issues. The secondary transformation of monoterpenes observed in spearmint may be largely due to the presence of endogenous enzymes that can promptly act on these introduced monoterpenes. In plants, oxidation of monoterpenes is largely carried out by cytochrome P450 oxygenases and glycosylation is mediated by UDP-glycosyltransferases (UGTs) [60,63,64,65]. From our previously published spearmint PGT specific transcriptomic data, we found that several cytochrome P450s and glycosyltransferase transcripts are highly enriched in PGT. Functional characterization of these will help identify the enzymes that catalyze the metabolism of these introduced monoterpenes. For successful establishment of heterologous plant platforms, the diverse array of reaction that modifies the primary products needs to be controlled. Genome editing approaches to knock out the endogenous enzymes responsible for modifications can limit the metabolism of heterologous products.

Our finding suggests that more in depth studies to understand all levels of regulation of metabolic pathway and its interaction with associated pathways in a specific plant type is necessary before it can be a chassis for producing new metabolites of interest. 

## 4. Materials and Methods

### 4.1. Plant Propagation and Samples Collection

Spearmint plants were grown in greenhouse under natural light and propagated by stem cuttings. Young leaves (2–3 cm) were used for gene expression analysis by q-PCR and for terpenoid profiling by GC-MS and LC-MS analysis. Chemicals such as methanol, acetonitrile, formic acid, ethyl acetate, camphor, linalool, myrcene, geraniol, limonene and carvone were purchased from Sigma-Aldrich (Singapore).

### 4.2. Vector Construction and Plant Transformation 

The *MsLS* RNAi construct was generated as follows. A 465-bp fragment of the *MsLS* exon and inverted repeat fragment amplified with suitable restriction enzymes were cloned into the donor vector and subsequently introduced into destination vector pK7WG2D by LR recombination. Binary vectors were introduced into *Agrobacterium tumefaciens* strain EHA105 and the spearmint plants were transformed by leaf disk transformation protocol [35]. Transgenic shoots were selected based on enhanced green fluorescence protein (eGFP) fluorescence. The eGFP positive plants were further confirmed by genomic amplification of RNAi cassette and Kanamycin marker gene and later by southern blot.

One single T-DNA insertion line with reduced endogenous monoterpene biosynthesis, referred as *MsLS* RNAi4 was chosen as a platform to overexpress the heterologous terpene synthases. Three monoterpene synthase genes were cloned and transformed into *MsLS* RNAi4, respectively. Coding sequence of linalool synthase (PaLinS) and myrcene synthase (PaMyrS) from *Picea abies* and geraniol synthase (CoGerS) from *Cananga odorata* were cloned into a gateway donor vector pENTR™/D-TOPO^®^ (Invitrogen) then introduced into destination vector pB7WG2D. Binary vectors were introduced into Agrobacterium tumefaciens strain EHA105, followed by leaf disk transformation. Transgenic plants were selected based on basta resistance and further confirmed by gene fragment sequencing and southern blot.

The RNAi cassette and the heterologous terpene synthases were driven by 35S promoter. Primers for *MsLS* RNAi fragment, the heterologous terpene synthases genes and qPCR sequence are listed in Supplementary Data (Figure S8).

### 4.3. DNA Isolation and Southern Blot

DNA was isolated by CTAB method as described [66]. After digestion with restriction enzymes EcoRI and XbaI, the DNA samples, 15 µg each lane, and the Dig labelled marker were separated by electrophoresis on 1.0% agarose gel and transferred to a Hybond-N+ nylon membrane (Amersham, Singapore). Primers used for amplification of NptII and bar gene are listed in Supplementary Data. NptII and bar gene fragments were used as probes for checking T-DNA insertion number for *MsLS* RNAi lines and lines expressing heterologous monoterpene synthases, respectively. DNA probe was generated by DIG probe synthesis kit and membranes were hybridized and washed according to a DIG DNA labelling and detection kit (Roche, Singapore). The hybridization bands were visualized by ChemiDocTM Touch Imaging System (Bio-Rad, Singapore).

### 4.4. RNA Extraction and Quantitative RT-PCR

Total RNA was extracted from leaf of spearmint using RNeasy^®^ Plus Mini kit from Qiagen (Singapore). About 1 µg total RNA was used to synthesize first strand cDNA. Reverse transcription reaction and quantitative RT-PCR were carried out by iScipt RT Supermix (Bio Rad, Singapore) and KAPA SYBR FAST qPCR Master Mix (2×) (Sigma-Aldrich, Singapore), respectively. Data analysis was performed using Relative Quantitation software from ABI using 2^−∆∆CT^ method. Expression of *MsLS* in RNAi lines are represented as relative to that of wild type (WT), where the expression of *MsLS* in WT is assumed as one hundred percent. Expression of heterozygous genes (*PaLinS*, *PaMyrS* and *CoGerS*) are represented as relative to ELF, a housekeeping gene elongation factor 1. The values in the graphs are the mean of three biological replicates and the error bars shows the standard deviation from mean values.

### 4.5. Protein Purification and In Vitro Enzymatic Assays

To express and purify recombinant MsLS protein for in vitro activity study, MsLS ORF was amplified and the fragment was cloned into pDEST-6His vector by gateway. The plasmid, carrying the open reading frame of MsLS was transformed into *E. coli* BL21 (DE3) (Invitrogen, Singapore). Positive clone was grown in LB medium at 37 °C to an OD_600_ of 0.4. Protein expression was induced with 0.5 mM IPTG and incubated at 22 °C, 200 rpm overnight. The 50 mL of cells harvested by centrifugation were resuspended in ice cold 1× PBS buffer with 1 mg lysozyme. The mixtures were sonicated 5 times for 20 s. Cleared lysate containing the protein was purified as described by Ni-NTA Agarose kit (Qiagen). Purified MsLS was used for in vitro enzymatic assay.

For terpene synthesis activity assay, 10 µg of purified MsLS was incubated with 20 ng/µL GDP in 25 mM HEPES (pH 7.4), 100 mM KCl, 10 mM MnCl_2_, 10% glycerol and 5 mM DTT. The 500 µL assay mixture was overlaid with 0.5 mL of ethyl acetate to trap volatile products. In order to test for sesquiterpenoid and diterpenoid activity, MsLS was incubated with 20 ng/µL FDP and GGPP in 25 mM HEPES (pH 7.4), 20 mM MgCl_2_, 100 mM KCl, 10% glycerol, 10 mM DTT for sesquiterpene assay and in 25 mM HEPES (pH 7.4), 10 mM MgCl_2_, 10 µM MnCl_2_, 100 mM KCl, 5% glycerol, 5 mM DTT for diterpene assay. All enzymatic reactions were incubated for 2 h at 30 °C. The reaction mixture was extracted with ethyl acetate twice. All the extracts were combined and were concentrated by nitrogen gas to 50 µL for analysis by GC-MS.

### 4.6. Volatile Compound Analysis by Gas Chromatography -Quadrupole MS (GC-MS) and Gas Chromatography Time-of-Flight Mass Spectrometry (GC-QTOF-MS)

About 100 milligrams of leaf was extracted with 500 µL of ethyl acetate containing 100 µg/mL of camphor (Sigma-Aldrich) as an internal standard. After 2 h incubation at room temperature with shaking at 120 rpm, the ethyl acetate fraction was transferred into a new tube and dehydrated by anhydrous Na_2_SO_4_. The ethyl acetate extraction was filtered through a 0.45 µm nylon filter and used for analysis.

Terpene products were identified and quantified by GC-MS analysis as described previously [21]. GC analysis was performed on a Agilent GC 7890A system equipped with HP-5MS GC column (30 m × 0.25 mm × 0.25 µm; Agilent Technologies, Singapore) using helium as the carrier gas. GC running conditions were as follows: the temperature program involved heating the oven at 50 °C for 1 min and then increased to 300 °C at 5 °C/min and kept at 300 °C for 5 min. The injector temperature was set at 230 °C with splitless injector mode. For each analysis, 2 µl sample was injected. The amount of each compound was calculated according to its peak area. Camphor was used as an internal standard. Compounds were identified by comparison of their mass spectra with those in the NIST MS 2014 library. For the identification of fatty acids, sterols and the volatile derivatives, a GC-QTOF-MS analysis was performed. About 500 mg of fresh leaves were extracted twice with total volume of 6ml of methanol and briefly vortexed, centrifuged for 10 min at 1500 g and filtered. The extracts were concentrated under flow of nitrogen and 1ul of concentrated extracts were used. GC-QTOF-MS was performed on Agilent GC-QTOF 7200B system equipped with an HP-5MS fused silica capillary column (30 m × 0.25 mm × 0.25 μ m) with helium as carrier gas. Oven was set at a temperature program of 50 °C for 1 min, increased at a rate of 8 °C/min to 300 °C, and held for 5 min. The detector temperature was 280 °C with a mass range from 45 to 450 mass-to-charge ratio, with electron energy of 70 electron volt. Identification of compounds were done by NIST library mass spectral database using the Mass Hunter data acquisition software. Quantification of the compounds were performed with the respective standards at different concentrations to establish a calibration curve. Quantification of compounds without available standards was carried out using the peak intensity for the relative percent composition. For each study, three biological replicates were used.

### 4.7. Non-Volatiles Compounds Extraction and Liquid Chromatography Quadrupole Time -of-Flight Mass Spectrometry (LC-QTOF-MS) Analysis

Analysis of non-volatile compounds in the respective transgenic plant extracts was performed using micrOTOF-Q^TM^ II LC system (Bruker Daltonics, Bremen, Germany) coupled with a mass spectrometer equipped with a dual electrospray ionization (ESI) source and MS workstation 8.2.1. Aliquots of 500 mg fresh leaves were extracted with 1.5 ml of 99.9% MeOH/0.133% formic acid in 2.0 mL Eppendorf tube. After short vortex and 15 min sonication, the extracts were centrifuged and filtered through 0.45 mm filter. The extracts were analyzed using micrOTOF-Q^TM^ II LC system coupled with a mass spectrometer equipped with a dual electrospray ionization (ESI) source and MS workstation 8.2.1. The UHPLC system included a binary pump, vacuum solvent degasser, autosampler with 108-vial well-plate trays, and thermostatically controlled column compartment. A reversed phase column, ChromolithPerformance RP-18e (2.0 × 100 mm, Merck, Kenilworth, NJ, USA.) was used and the mobile phase comprised of water with 1.0% formic acid and 10 mM ammonium acetate (A) and methanol (B) with a flow rate of 0.3ml/min. The linear gradient elution was programmed as 10.0 min, 5% B; 0 min, 5% B; 7 min, 50% B; 10 min, 95% B; 20 min, 95% B; 21 min, 5% B; 25 min, 5% B. The spectra were acquired in the negative ion mode over a mass-to-charge (*m*/*z*) range from 50 to 1200 amu. The data obtained from the LC-QTOF-MS/MS runs was processed through the Data Analysis 4.0 software (Bruker Daltonics, Bremen, Germany). The features from the data sets was further processed using Bruker Compass Profile Analysis 2.1 software to analyze LC-MS data. Molecular formula determination was carried out by mass accuracy and fragmentation information using the smart formula. For multiple possible molecular formulas (tolerance up to 5ppm) the best matches were searched in the METLIN and PUBCHEM databases for possible structures, where compounds were identified based on their retention time, mass, and MS/MS fragments. Finally, the identification and the quantitative results obtained from the data analysis was used for the statistical analysis. For quantification of the metabolites, a calibration curve with a series of standard solutions was performed and the compounds were quantified based on the relative response for the amount produced in the plant.

### 4.8. Statistical Analysis

Data are expressed as the mean ± SD of three replicates. Statistical significance between transgenic plants and wild type was analysed using a two-tailed Student’s *t*-test as indicated by asterisks. For better view the data, we made whisker plot for Figure 2B and Figure 3A,B (Supplementary Figure S9). SPSS statistical package software (SPSS for Windows version 20, USA) was used for the statistical analysis of the data. Principal component analysis (PCA) technique using SIMCA-P software, version 9 (Umetrics) was used for the dimensionality reduction of the data to present the samples in a new coordinate system to reveal the differences in the metabolic composition.

## Figures and Tables

**Table 1:** Compounds that were found significantly increased in MsLS RNAi lines by LC-MS analysis in both negative and positive mode.

**Compounds**	**Retention Time (min)**	**Mass**	**Molecular Formula**	**MS/MS Fragment**
Vanillyl mandelate *	5.30	197.01	C9H9O5	197,315
*cis*-Dihydroxycinnamate	12.90	179.03	C9H7O4	179,353
Ferulic acid	19.97	193.02	C10H9O4	193,387
Isovitexin β-d-glucoside	23.65	593.15	C27H29O15	593,433
Kaempferol-3-O-d-glucoside	24.01	447.09	C21H19O11	447,221
Salvianolic acid	49.91	493.00	C26H22O10	295,185
Isocoumarins	45.60	553.13	C28H25O12	553,359
Trihydroxymethoxy isoflavonone *	36.07	301.07	C16H13O6	301,359
Cinnamyl 6-O-(α-L-rhamnopyranosyl)-β-D-glucopyranoside *	28.12	503.17	C22H31O13	503,221
Megastigmane glucoside*	45.71	401.18	C19H29O9	401,221

Asterisk denotes new compounds identified in MsLS RNAi lines.

**Table 2:** Compounds that were found significantly increased in MsLS RNAi lines by GC-MS analysis.

Class of Compounds	Putative Identity	Retention Time (min)	Fragment Ion (m/Z)
Sesquiterpenes	Caryophyllene	13.75	93
	*cis*-β-Farnesene	15.50	41
	*cis*-Muurola-4(15),5-diene	14.50	161
	Germacrene D	14.90	161
Fatty Acids	Methyl palmitate	19.18	74
	Methyl stearate	20.91	74
	Linolenyl myristate	20.87	474
Steroids	Sitosterol	21.50	43
	Stigma sterol	22.63	55

**Table 3:** Putative geraniol derivatives, linalool derivatives and myrcene derivatives identified by LC-MS analysis in both positive and negative mode.

Transgenic Lines	Compounds	Retention Time (min)	Mass	Molecular Formula	MS^n^ Fragment
Geraniol derivatives	Hexosyl hydroxygeranic acid	36.77	345.15	C16H25O8	179, 183, 327,331
	Malonyl-hexosylgeraniol dimer	45.71	803.36	C38H59O18	221, 401, 643, 717
	Malonyl hexosylgeraniol acid dimer	51.44	831.36	C38H55O20	191, 349, 371, 415
	Loganin	13.90	391.16	C17H27O10	193, 461, 517, 691
Linalool derivertives	Linalool-3-glucoside	8.69	316.39	C16H28O6	71, 93, 55
	Linalool butyrate	49.52	224.34	C14H24O2	93, 69, 121
	Linalyl β-D-glucopyranoside	40.32	315.18	C16H27O6	163, 444, 607
Myrcene derivatives	Myrcenyl acetate	24.82	196.26	C12H20O2	395, 537, 59
	Dimethyl-3-oxopentane-1-5 diyl dibenzoate	49.46	221.18	C13H17O3	289, 407, 465

## Supplementary Materials

Supplementary materials can be found at https://www.mdpi.com/1422-0067/21/17/6164/s1.
