# Supplementary figures and images for: Metabolic Engineering of the Native Monoterpene Pathway in Spearmint for Production of Heterologous Monoterpenes Reveals Complex Metabolism and Pathway Interactions
Source: Int J Mol Sci. 2020 Aug 26;21(17):6164. doi: 10.3390/ijms21176164 (PMC7504178; doi:10.3390/ijms21176164)

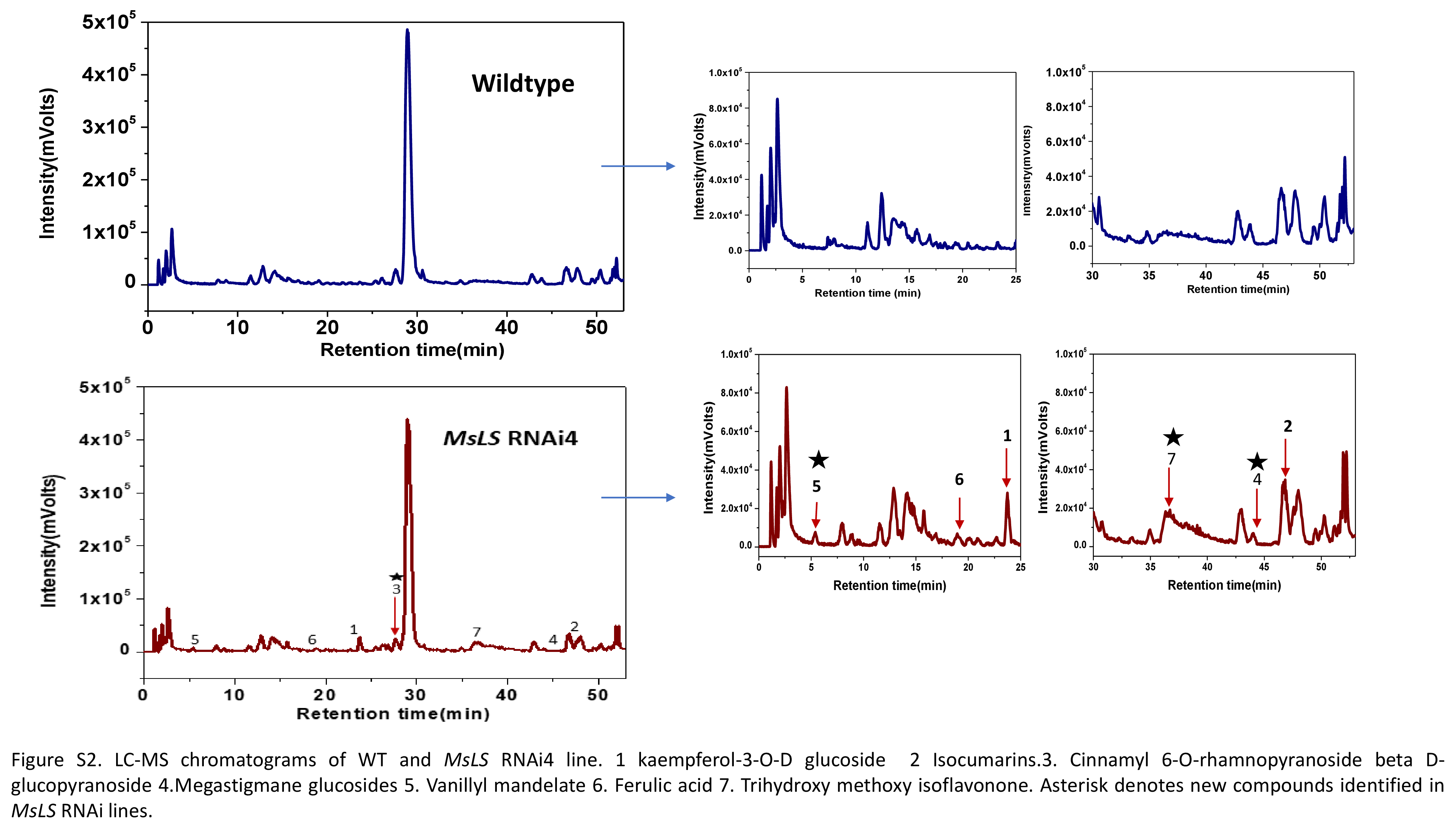

Supplement: Supplementary file 1 [file ijms-21-06164-s001.zip › final Supplementary figures/supplementary figure2.tiff]

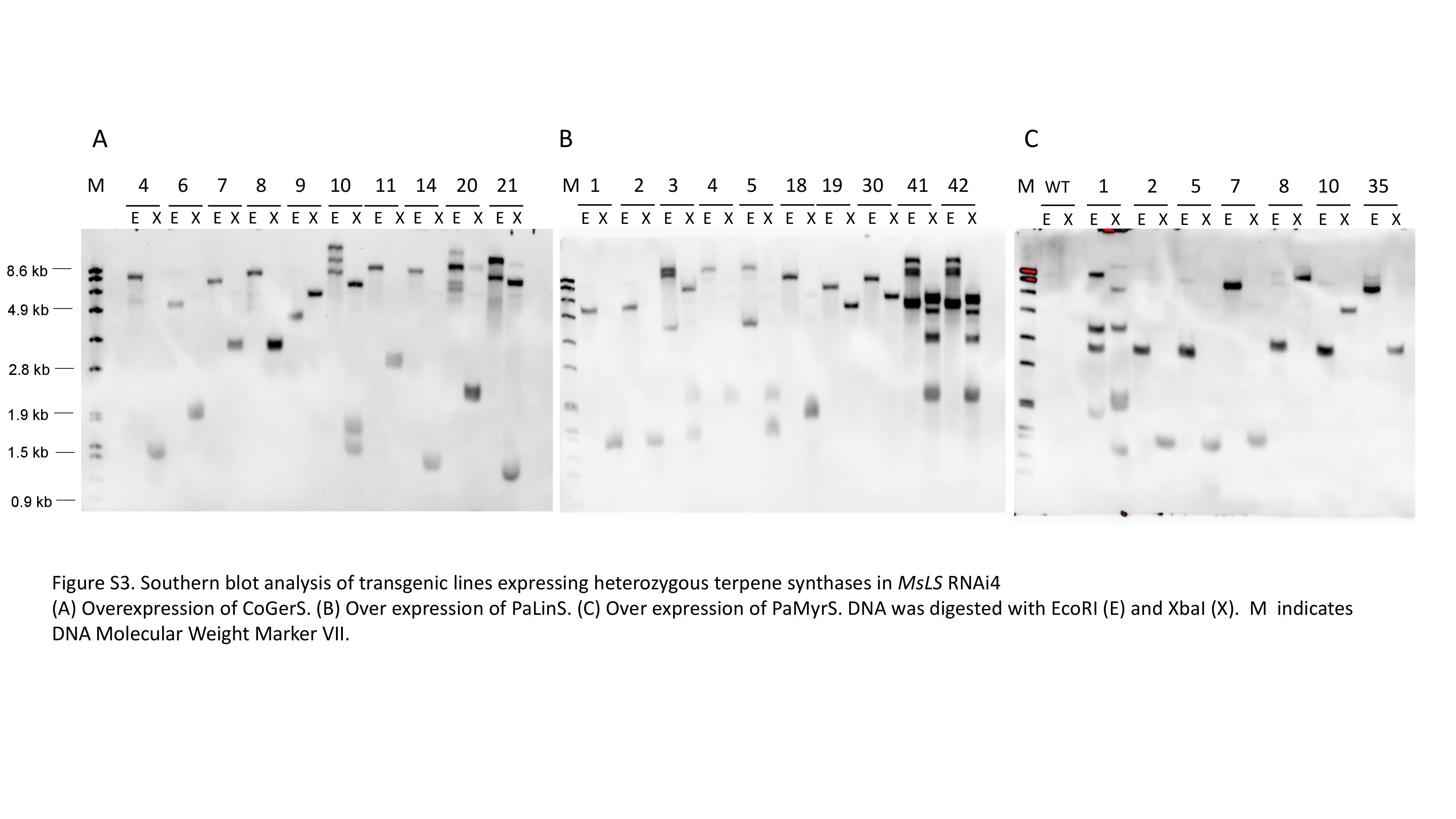

Supplement: Supplementary file 1 [file ijms-21-06164-s001.zip › final Supplementary figures/supplementary figure3.tiff]

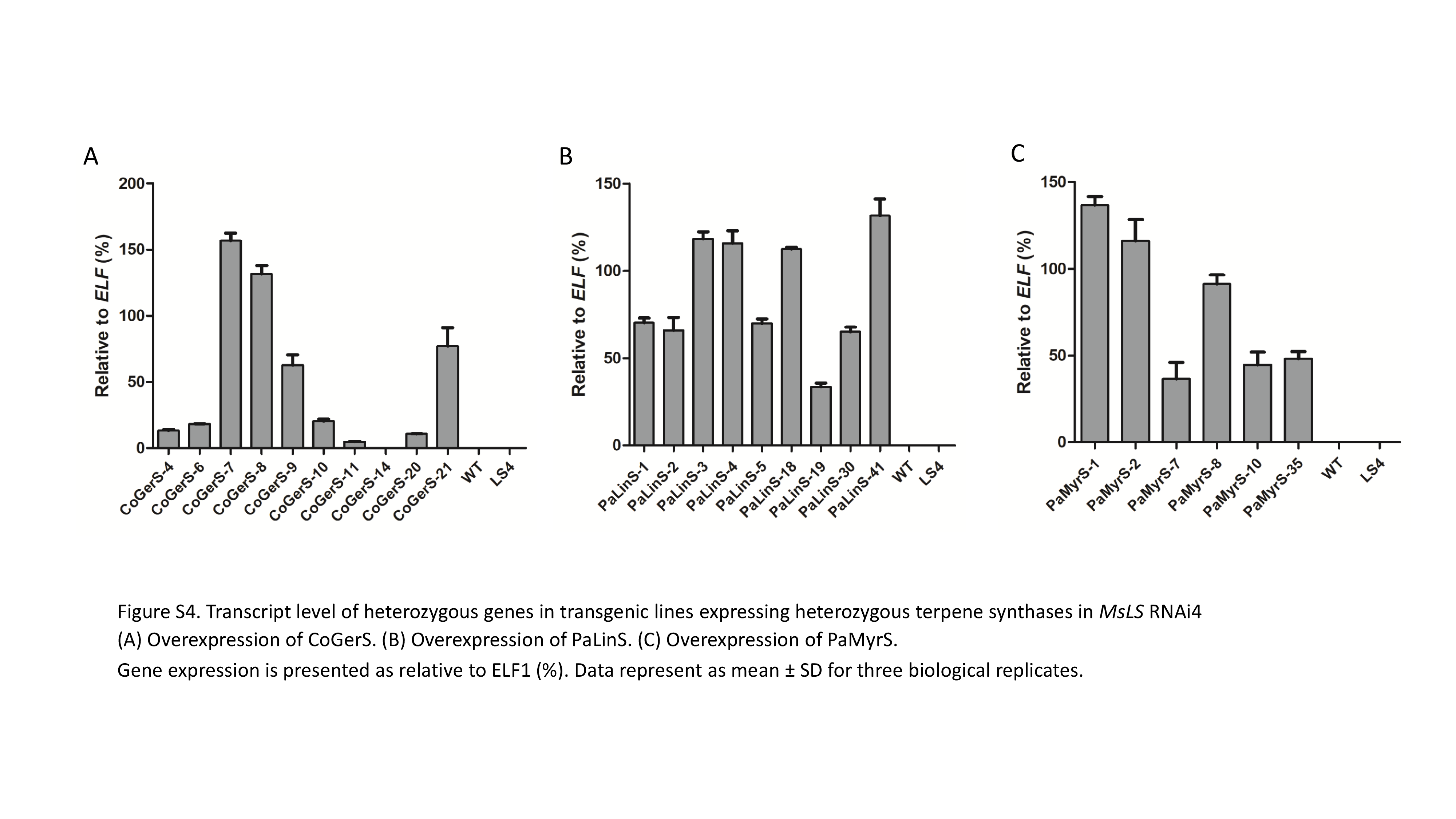

Supplement: Supplementary file 1 [file ijms-21-06164-s001.zip › final Supplementary figures/supplementary figure4.tiff]

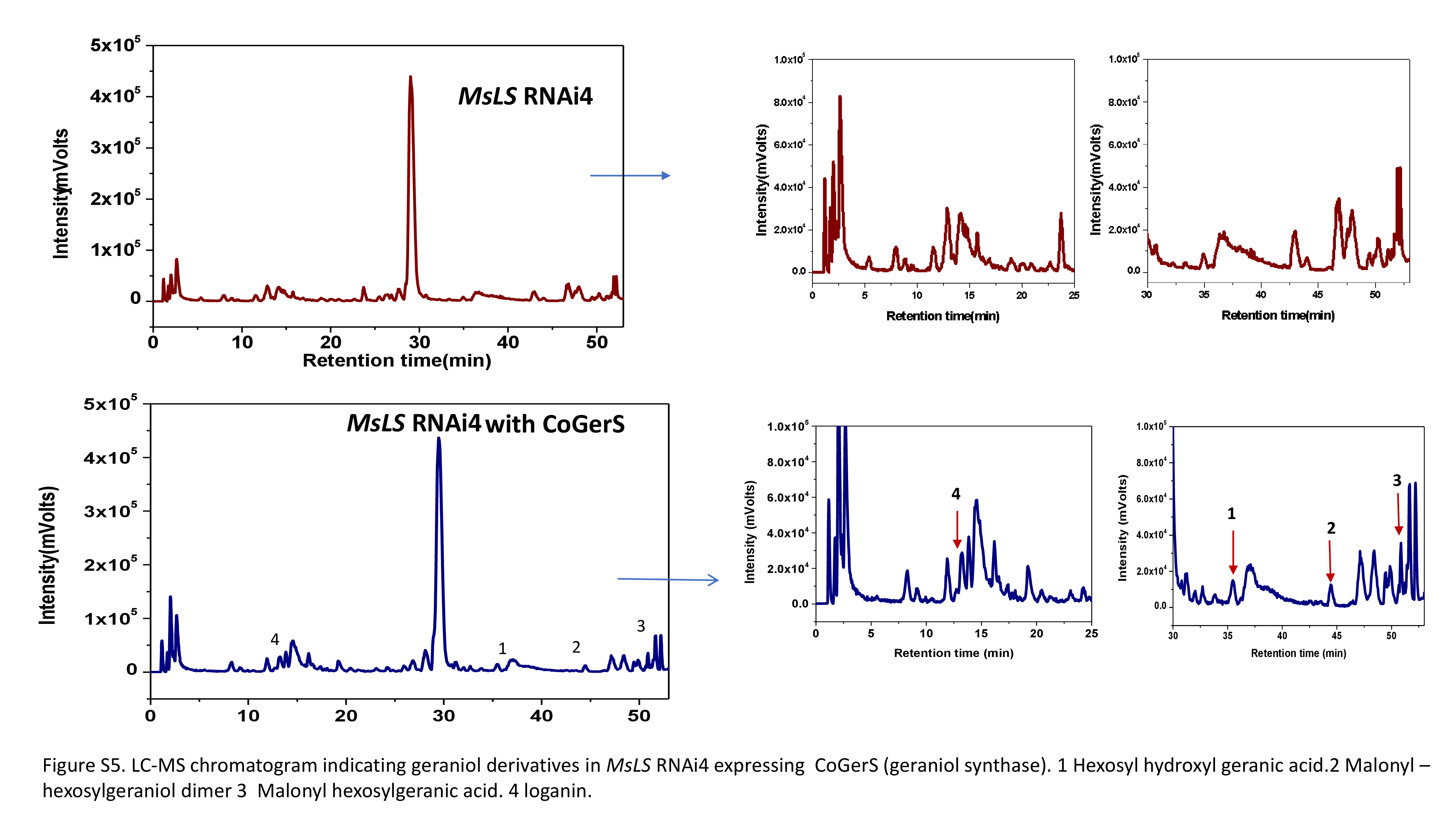

Supplement: Supplementary file 1 [file ijms-21-06164-s001.zip › final Supplementary figures/supplementary figure5.tiff]

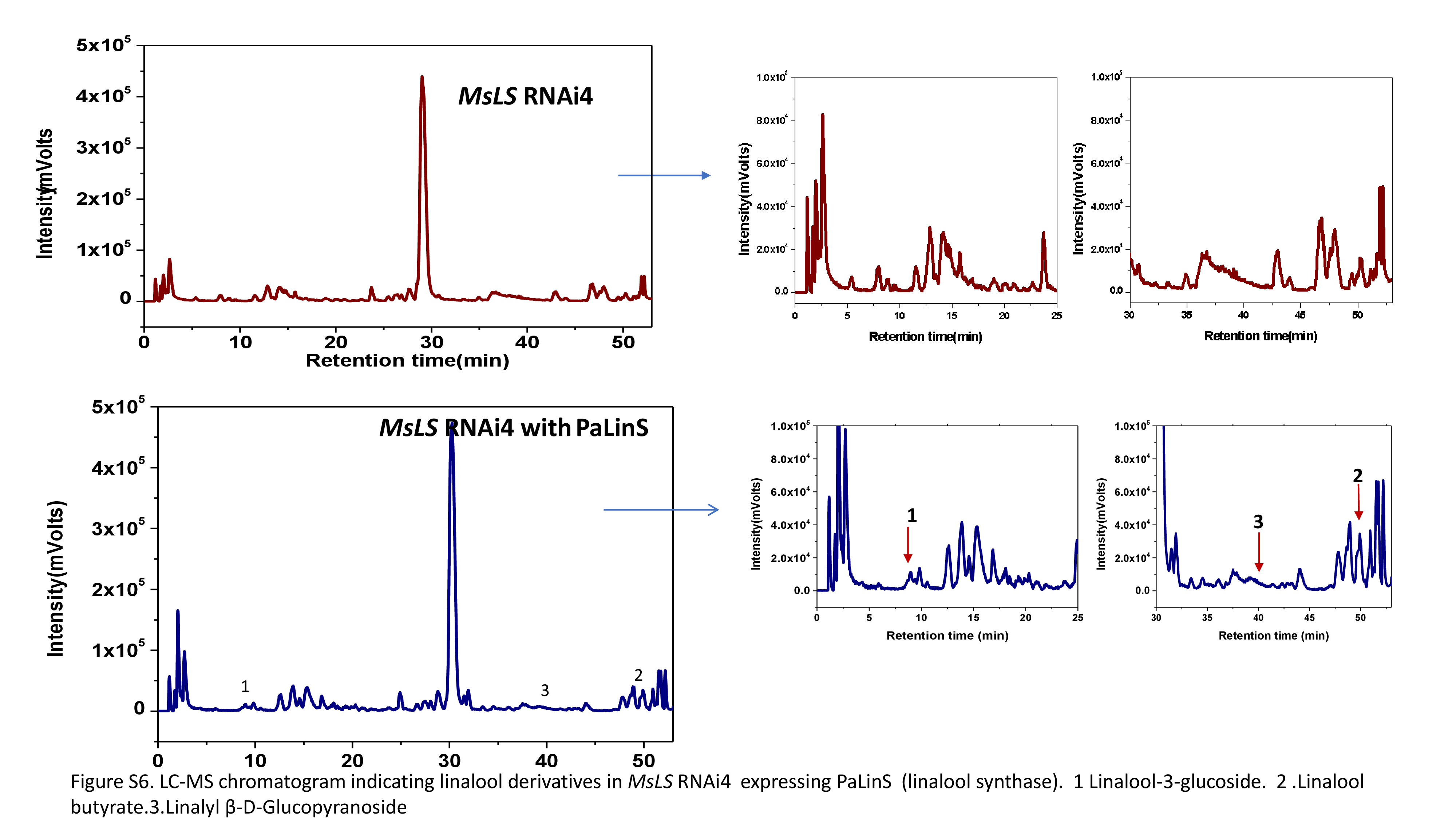

Supplement: Supplementary file 1 [file ijms-21-06164-s001.zip › final Supplementary figures/supplementary figure6.tiff]

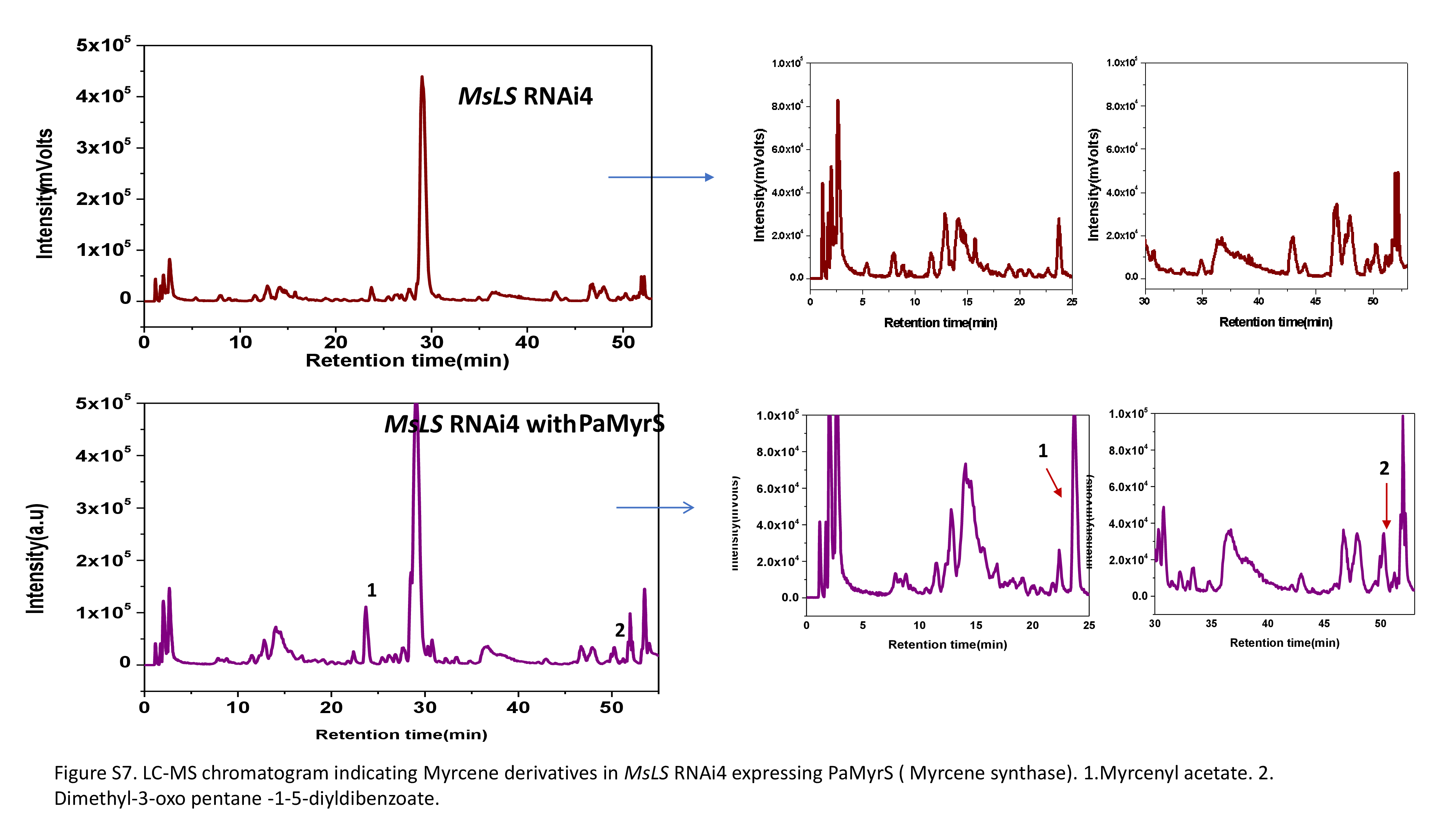

Supplement: Supplementary file 1 [file ijms-21-06164-s001.zip › final Supplementary figures/supplementary figure7.tiff]
